# Confirmatory Factor Analysis of the Enriched Life Scale Among US Military Veterans

**DOI:** 10.3389/fpsyg.2019.02181

**Published:** 2019-10-11

**Authors:** Caroline M. Angel, Mahlet A. Woldetsadik, Justin T. McDaniel, Nicholas J. Armstrong, Brandon B. Young, Rachel K. Linsner, John M. Pinter

**Affiliations:** ^1^Team Red, White & Blue, Alexandria, VA, United States; ^2^Reintegrative Health Initiative, Westfield, NJ, United States; ^3^Institute for Veterans and Military Families, Syracuse University, Syracuse, NY, United States; ^4^Pardee RAND Graduate School, Santa Monica, CA, United States; ^5^Department of Public Health and Recreation Professions, Southern Illinois University, Carbondale, IL, United States; ^6^Tennyson Center for Children, Denver, CO, United States

**Keywords:** Enriched Life Scale, confirmatory factor analysis, veteran, wellbeing, Team Red, White & Blue, psychometric assessment

## Abstract

The Enriched Life Scale (ELS) is a 40-item measure developed by the military veteran service organization, Team Red, White & Blue (RWB), to systematically capture and quantify the lived experiences of military veterans transitioning to civilian life. As Team RWB’s mission is to “enrich veterans’ lives,” veterans who conceived of and co-developed the ELS as a psychometric instrument defined what an “enriched life” would entail. Exploratory factor analysis (EFA) of the ELS revealed a five-factor structure capturing the domains of: physical health, mental health, genuine relationships, sense of purpose, and engaged citizenship. The goal of the current study was to use confirmatory factor analysis to validate the factor structure of the ELS in a sample of veterans not affiliated with Team RWB. We also sought to explore convergent validity with the Military to Civilian Questionnaire, a measure of military to civilian reintegration challenges. Five hundred and twenty-nine veterans participated in the study. We estimated three models, one-factor, four-factor, and five-factor model via maximum likelihood estimation with robust Huber-White standard errors. The five-factor model showed the best fit to the data (RMSEA = 0.05, CFI = 0.90, TLI = 0.90, SRMR = 0.06). Additionally, the five-factor model demonstrated convergent and discriminant validity, as well as internal consistency reliability (genuine relationships, α = 0.90; sense of purpose, α = 0.93; engaged citizenship, α = 0.89; mental health, α = 0.88; and physical health, α = 0.78). Overall, the ELS is a valid and reliable measure of veteran enrichment and could potentially be used in conjunction with diagnostic instruments that capture strain-related transition challenges (to include mental health disorders) to capture post-military service wellbeing.

## Introduction

Military veterans must navigate a range of challenges in their transition to civilian life. While the transition from service member to veteran is primarily characterized by resilience, many veterans experience lasting physical, psychological, and social problems related to military service and reintegration (Angel, 2016; Elnitsky et al., 2017; Mobbs and Bonanno, 2018). Team Red, White & Blue (RWB) was founded in 2010 to offset service-related reintegration stressors by providing opportunities for veterans to connect with service-connected peers and civilian community members. Over 200 national chapters create local and consistent opportunities for members to participate in physical, social, leadership, and volunteering activities. In 2018, Team RWB’s membership reached over 153,000 members and over 2,000 volunteer leaders created 38,000 Team RWB events^1^. The mission of Team RWB is to “enrich veterans’ lives” and the foundational veteran thought leaders spent years developing this theoretical model of engagement and defining what it means to “enrich” a life. Leaders ultimately defined an “enriched life” as having physical, mental, and emotional health; genuine relationships comprised of close, best-friend types of relationships within a broader social network; and a sense of purpose, which included an individual sense of purpose, shared purpose, and positive role identity (Angel et al., 2018a).

Team RWB was founded in 2010 by Army Captain, Michael S. Erwin, who was studying positive psychology principles under the field’s co-founder, Christopher Peterson. Positive psychology focuses on “what goes right in life” (Seligman and Csikszentmihalyi, 2000); Team RWB was established to connect transitioning veterans to their community through activities that supported physical activity and helped them develop and maintain personal and community connections (Angel and Armstrong, 2016). With increasing negative health behaviors and weight gain as major issues affecting veterans along with the loss of camaraderie, sense of purpose, and shared mission with others, Team RWB was filling a gap by offering a new approach to supporting transitioning veterans to their communities (Angel et al., 2018a). While Team RWB was leveraging the principles of positive psychology for community dwelling veterans, the movement of positive psychology had just begun to rise in the Army itself. In 2008, the Army implemented the Comprehensive Soldier Fitness Program, designed to increase active duty soldiers’ psychosocial and positive performance through assessment and training. As physical fitness tests were already routinely in place, Army leaders were proactively developing soldier psychosocial resilience thereby hoping to decrease psychological disorders as a result of military service (Cornum et al., 2011). As *resilience* became the focus of the Army’s positive psychology training program, Team RWB leaders purposely avoided language reminiscent of active duty service, which they believed would be off putting to new members who were recently transitioned out of the service, and may wish to avoid that reminder. “Enriching lives,” ultimately most resonated with Team RWB’s founder more so than other concepts of well-being, to which it is highly related (Angel and Armstrong, 2016).

In the extant literature, the concept of an “enriched life” is theoretically related to constructs such as well-being, life satisfaction, and flourishing (Angel et al., 2018a). We have previously described how conceptualizations of “veteran wellness” broadly defined as satisfactory function in the areas of personal relationships, health, fulfillment of material needs, and having a sense of purpose is applicable to veterans and civilians alike (Angel et al., 2018a). More traditional conceptualizations of well-being, however, have traditionally neglected the physical health component. Ryff’s (2018) foundational definition of “well-being” was formulated based upon the philosophical tenets first articulated by Aristotle and developed by psychologists from clinical, developmental, humanistic, existential, and social perspectives. Ryan and Deci (2001) defined well-being as optimal psychological functioning and experience organized by two central perspectives: hedonic and eudaimonic well-being. The hedonic approach focuses on pleasure seeking and pain avoidance for body and mind while the eudaimonic approach focuses on meaning and self-actualization. In the eudaimonic tradition, Ryff developed a theory-guided measure of psychological well-being. The widely used measure, the Ryff Scales of Psychological Well-Being assessed six constructs: self-acceptance (positive attitude toward the self), positive relations with others (warm, satisfying, trusting relationships with others), autonomy (self-determining/independent), environ-mental mastery (competence in managing the environment), purpose in life (direction and meaning in life), and personal growth (feelings of continued development) (Ryff, 1989). Veteran conceptualization of their own well-being is aligned to eudaimonic approaches, integrating a sense of purpose and opportunities to serve others through volunteering and leading others as key components.

“Life satisfaction” has been deemed a cognitive component of subjective well-being, described as a general self-appraisal of one’s own quality of life (Pavot and Diener, 2009). It’s most widely used measure, the Satisfaction with Life Scale (Diener et al., 1985), is unidimensional, capturing the factor of “life satisfaction” which is theoretically related to an enriched life. Finally, the concept of “flourishing” has been defined as having positive emotion, engagement, relationships, meaning and accomplishment (Seligman, 2011). While more recent conceptualizations of flourishing published following the development of the Enriched Life Scale (ELS) have included references to positive physical health (VanderWeele, 2017), traditional conceptualizations of flourishing have primarily overlooked physical health as a key component.

Team RWB leaders explored a variety of existing instruments prior to the development of the ELS. Scales that have measured well-being have trended to capture between one to six constructs on the dimensions of well-being: global well-being, social well-being, physical well-being, spiritual well-being, activities and functioning, and personal circumstances, and run between five and one hundred or more items (Linton et al., 2018). Linton et al.’s (2018) review of 99 self-report measures for assessing wellbeing in adults describes these instruments in depth. While Team RWB leaders admittedly did not examine every instrument reviewed by Linton et al. (2018) prior to the development of the ELS in 2014, they believed the original enrichment equation (five constructs to include physical health; mental health; emotional health; genuine relationships; and sense of purpose) would need to measure all domains that they felt captured veterans’ lived experience of an enriched life and was detailed enough to provide information back to the organization so that Team RWB leaders could actively engage members through specific, needs-driven (potentially individualized) activities. Therefore, driven by their operational experience of designing and deploying survey instruments in a non-profit membership environment, for which the ELS was originally developed, they hypothesized that the instrument should be between 25 and 45 items. Existing instruments considered, like the Ryff Scales of Psychological Well Being (Ryff, 1989), the Perma Profiler (Butler and Kern, 2016), the Flourishing Scale (Diener et al., 2010), were deemed too narrow in scope theoretically or too short to adequately capture what veteran leaders felt defined an “enriched life”. Other instruments provided simple yes/no checklists yielding too limited information to provide operationally useful feedback (Linton et al., 2018). Additionally, at least two widely used scales, the Conner Davidson Resilience Scale (Green et al., 2014), and the Perma Profiler (Butler and Kern, 2016) have demonstrated new factor structures differing from the original when tested in veteran populations (Umucu et al., 2019).

Veteran leaders and consulting academics also considered the translational capabilities of existing measures. They viewed the translation of the constructs of other popular instruments to a broader lay-person public health communication strategy as limited. The concepts themselves are semantically representative of academic terminology and would be lost on an audience unfamiliar with such discipline-specific terms (for example, “environmental mastery”). Often they found the terminology lacking cultural congruity to veteran serving community based organizations, in which communications are more generally guided by marketing, development, and personal relations professionals than researchers or clinicians. Even the U.S. Army developed Global Assessment Tool, an assessment of soldier psychosocial fitness tailored to the Comprehensive Soldier Fitness Program, did not assess physical health component, which is fundamental to Team RWB’s mission; given its 105 item length, it could not feasibly be administered to newly joining members of the community based veteran service organization.

Therefore, Team RWB veteran thought leaders and social scientists spent three years (2014–2017) developing the ELS (Team Red White Blue, 2017), which was finalized as a 40-item instrument in 2017 (Team Red White Blue, 2017; Angel et al., 2018b). The need for an instrument with valid and reliable psychometric purposes was driven by Team RWB’s desire to be accountable and transparent to key stakeholders (members, funders, supporters) in their articulation and measurement of the impact of their programs in achieving their stated mission. Additionally, the ability to provide a veteran-developed assessment tool which placed veterans’ needs and lived experiences of transition from military to civilian life as the guiding voices in determining successful transition filled an assessment and research gap. It also permitted the development of an instrument that could feasibly be administered to thousands of newly joining Team RWB members, which Team RWB is currently exploring.

Preliminary psychometric properties were established for the 40-item ELS in a sample of 1,187 military veterans and 598 civilians, all members of Team RWB (Angel et al., 2018b). The theoretical model of an “enriched life” was mostly validated, with the exception that the hypothesized construct, “emotional health” did not emerge as a stand-alone construct. Instead, items originally written to reflect the definition and measurement of “emotional health” fell under the “genuine relationships” or “sense of purpose” constructs. Additionally, items written to reflect the “sense of purpose” construct emerged as a new factor, which authors labeled “engaged citizenship”. Engaged citizenship was subsequently defined as “the sense of belonging and responsibility to a larger community that promotes altruistic behavior through leadership and civic action”. Engaged citizenship is culturally authentic to veterans, many of whom seek and value opportunities for community service and leadership during their transition from military to civilian life. Veteran and civilian ELS factors were identical, except for one sleep-related item, which loaded onto physical health for the mostly female civilian sample, and mental health for the mostly male veteran sample. Civilians scored higher on every subscale of the ELS and total score than veterans, with small to medium effect size differences. In the veteran sample, veterans with combat experience and service-related injuries scored lower on the ELS than veterans without combat experience or service related injuries. As the ELS was preliminarily validated in a sample of Team RWB members, the inherent bias was that members may have already been exposed to life enriching activities via participation in the organization, although the preliminary study was not designed to serve as a program evaluation framework for Team RWB. In the current study, we tested the ELS factor structure in a sample of non-Team RWB members to potentially increase generalizability to other populations of veterans; we were uncertain if veteran Team RWB members shared an inherent bias that our methods were not sensitive enough to detect when they self-selected into a fitness and social activity focused organization. Additionally, while the development and implementation of the ELS is to measure an enriched life in veterans and civilians, we limited the current study to veterans as it was the most highly prioritized need for Team RWB as veterans are an understudied population and should thus be preferred.

The goal of the current study was to use confirmatory factor analysis to validate the structure of the ELS in a sample of veterans self-identifying as not affiliated with Team RWB. Our second objective was to explore convergent validity with the Military to Civilian Questionnaire (M2C-Q), a psychometric measure of reintegration difficulties in veterans (Sayer et al., 2011). We hypothesized that as veteran participants reported higher levels of enrichment, they would report lower levels of reintegration difficulties.

## Materials and Methods

### Participants

Participants were recruited electronically via direct email, partner Twitter and Facebook solicitations, and snowball sampling between March 2017 and March 2018 for a multi-purpose study. After providing informed consent, participants were directed to a secure link. Respondents self-identified as being a veteran, active duty military service members, or having no military service experience (civilians). A week after the original email was circulated, a reminder was sent to participants. A total of 1,900 respondents agreed to participate in the study through the recruitment period. Participants who were retained as part of this analysis were military veterans who self-reported that they were not members of Team RWB. We removed 800 participants from the analysis who reported that they were members of Team RWB and 78 participants who did not indicate whether they were members or not. This procedure was used in order to isolate the confirmatory factor analysis to non-Team RWB members, whom we hypothesized, may have already received life-enriching activities, based upon their exposure to Team RWB activities at the time of recruitment for the exploratory factor analysis (EFA) study (Angel et al., 2018b). Since the EFA was conducted on a sample of Team RWB members, the CFA was limited to non-Team RWB members in order to avoid overly optimistic model fit. In addition, 96 participants who started the survey but did not complete the ELS portion of the survey were removed from the analysis. Out of the remaining 926 participants, only U.S. military veterans were retained, resulting in a final sample size of 529 veterans for the CFA analysis. Each observation had complete data.

The study protocol was reviewed and approved by the Institutional Review Board at Syracuse University. Participants were informed that the purpose of the study was to develop a new instrument to track health, relationships, and sense of purpose. The average time to complete the entire 110-question survey, inclusive of the 40-item ELS, demographic variables, and other variables of interest to Team RWB, was 24 min. Qualtrics estimated that the 40-item ELS would take 8–9 min to complete by itself. No financial compensation was provided for completing the survey.

### Measures

The ELS (Team Red White Blue, 2017) is a 40-item measure that assesses “enrichment,” defined as physical health (having consistent physical activity, with appropriate restful sleep, nutrition, healthy weight maintenance, strength, and mobility to accomplish activities of daily living with ease); mental health (anxiety and depressive symptoms within normal limits to include controlled anger and an ability to focus, make decisions, and remember things); genuine relationships (a combination of weak and strong social ties that include close, “best-friend” types of relationships as well as a broader supportive network to provide emotional support, information, and resources); a sense of purpose (individual and shared goal driven activities integrated with positive emotion (optimism, gratitude, self-compassion, pride, open-mindedness) and positive role identity); and engaged citizenship (the sense of belonging and responsibility to a larger community that promotes altruistic behavior through leadership and civic action). ELS subscale length and example items of each scale are as follows: “genuine relationships” (11 items), “I have people in my life that are not my relatives but feel like family”; “sense of purpose” (12 items), “I have a sense of direction in my life”; “engaged citizenship” (6 items), “I feel like a leader in my community”; “mental health” (6 items), “Even when I feel nervous, anxious, or irritable, I am able to carry out day-to-day activities and responsibilities in my work and relationships,”; and “Physical Health” (5 items), “I have the strength and mobility to do all the things I need to do routinely in my life with ease”. With the exception of one four-point Likert scale (i.e., item #36) that assesses the frequency, duration, and intensity of physical exercise, all items were rated on a five-point scale in increments of 25 points (ranging from zero to 100), where higher scores indicated greater enrichment.

The Military to Civilian Questionnaire (M2C-Q) (Sayer et al., 2011) is a publicly available 16-item measure that assesses veterans’ post-deployment community reintegration difficulties. Areas assessed include (a) interpersonal relationships with family, friends, and peers; (b) productivity at work, in school, or at home, (c) community participation; (d) self-care; (e) leisure, and (f) perceived meaning in life. Items are rated on a 5-point Likert scale with these response options: 0 = No difficulty, 1 = A little difficulty, 2 = Some difficulty, 3 = A lot of difficulty, and 4 = Extreme difficulty. Respondents can indicate “Does not apply” for the four items that assess relationship with spouse/partner, relationship with child/children, work, and school functioning. The measure was validated in a study of 745 Iraq and Afghanistan veterans who sought medical care from the U.S. Department of Veterans Affairs (Sayer et al., 2011). The instrument was selected for convergent validity in military veterans, as we expected ELS subscales (ranging zero to 100) to be inversely related to the M2C-Q score on the basis that the ELS measures reintegration enrichment and the M2C-Q measures reintegration challenges.

### Statistical Analyses

For the confirmatory factor analysis of the ELS, three models were estimated via maximum likelihood estimation with robust Huber-White standard errors (MLR) (Li, 2018), as the assumptions for standard maximum likelihood estimation (i.e., multivariate normality) were not met. Based upon the findings of the EFA (Angel et al., 2018b), a one-factor model, where all 40-items of the ELS were arranged within one latent factor, was estimated first. We then tested a four-factor model, where “sense of purpose” and “engaged citizenship” were collapsed. Then, a five-factor model was estimated, including the following constructs and items: “genuine relationships” (GR, items 1–11); “sense of purpose” (SP, items 12–23); “engaged citizenship” (EC, items 24–29); “mental health” (MH, items 30–34); and “physical health” (PH, items 35–40). The three models were compared by examining the proportion of variance accounted for, the rotated loading patterns, and the Akaike information criterion (AIC) and the Bayesian information criterion (BIC), where smaller values indicated better fit (Burnham and Anderson, 2004). The key model fit statistics for the one-factor, four-factor, and five-factor models are shown in Table 2. Consistent with the findings of our EFA (Angel et al., 2018b), the five-factor model resulted in being the best fit. Residual correlations between items within the same construct were added iteratively to the five-factor model based on modification indices to improve model fit (Kline, 1998). This approach, described by Sorbom (1989) as the *post hoc* model modification approach or *post hoc* method theory, allows researchers to identify areas of theoretical misspecification within confirmatory factor analysis models, make adjustments to the theoretical model via consideration of modification indices, and generate more robust models. While there is some debate about the utility of this approach (Pan et al., 2017), we specified correlated residuals on within-construct items with modification indices greater than five (Segers, 1997) until satisfactory model fit was achieved (Sass, 2011). Residual correlations were also added to the following items due to item wording effects, such as parallel or negative wording, or item context, such as questions which reference a similar context (Schreiber et al., 2010; Asparouhov et al., 2015): GR 2 and 3; GR 3 and 10; SP 16 and 17; SP 19 and 20; SP 12 and 13; SP 18 and 20; SP 14 and 15; EC 28 and 29; EC 26 and 27; EC 25 and 28; MH 31 and 32.

Several indicators of model fit were used: the model Chi-square statistic, the Root Mean Square Error of Approximation (RMSEA), the Comparison fit index (CFI), the Tucker-Lewis fit index (TLI), and the Standardized Root Mean Square Residual (SRMR). Values of RMSEA ≤ 0.06, CFI/TLI ≥ 0.90, SRMR ≤ 0.10, and a *p*-value for the χ^2^ < 0.05 are often considered as indicating acceptable fit (Hu and Bentler, 1999; Mehmetoglu and Jakobsen, 2016). Convergent validity for the subscales was assessed by (a) estimating composite reliability (CR) for each factor, where a CR value >0.70 was considered evidence of convergent validity (Hair et al., 2008; Thornton et al., 2014), (b) examining factor loadings for statistical significance at an alpha level of 0.05 (Cole, 1987), and (c) by correlating factor scores from the validated five-factor ELS model with factor scores from the M2C-Q (Sayer et al., 2011), a measure that is theoretically inversely related to the ELS. For the purposes of standardizing comparisons between mean scores and standard deviations between the M2C-Q and the ELS, we recoded the M2C-Q response scale to correspond to the ELS (0, 25, 50, 75, 100). Discriminant validity within the five-factor ELS was assessed by calculating heterotrait-monotrait ratios of correlations (HTMT) among the five factors (subscales), using a criterion of <0.85 to indicate discriminant validity (Henseler et al., 2015). According to Henseler et al. (2015), the HTMT for two constructs is the average of the heterotrait-heteromethod correlations relative to the average of the monotrait-heteromethod correlations, as derived from the classic multitrait-multimethod matrix. We also assessed internal consistency reliability with Cronbach’s alpha and adopted a criterion of >0.70 to indicate reliability (Nunnaly, 1978). All analyses were conducted with the “lavaan” (Rosseel et al., 2018) and “semTools” (Jorgensen et al., 2018) packages within the R project for statistical computing (R Core Team, 2019).

## Results

### Participant Characteristics

Table 1 displays demographic characteristics for the sample which included a total of 529 veterans. Over 78% of veterans in our sample were male, and 60% of the sample was between the ages of 40 and 60, while 30% of veterans were younger than 40. Almost 80% of the sample was married or in a partnership and over 77% of the veterans had at least an undergraduate college education. Sixty-three percent of veterans were employed, while 11% were unemployed.

**TABLE 1:** Demographic characteristics of the study sample (*n* = 529).

	***n***	**%**
**Gender**		
Male	416	78.60
Female	113	21.40
**Age**		
20 to 40	167	31.60
41 to 60	321	60.70
61 to 80	32	6.00
Refused	9	1.70
**Race/Ethnicity**		
American Indian/Alaskan Native	5	0.90
Asian/Pacific Islander/Native Hawaiian	21	4.00
Black/African American	89	16.80
Hispanic	44	8.30
White	355	67.10
Other	13	2.50
Refused	2	0.40
**Employment Status**		
Employed	334	63.14
Unemployed	63	11.91
Retired	35	6.62
Student	46	8.67
Disabled	12	2.28
Other	39	7.38
**Marital Status**		
Single	54	10.21
Married/Partnership	420	79.39
Divorced/Separated	50	9.45
Widowed	2	0.38
Refused	3	0.57
**Educational Attainment**		
High School Degree	6	1.13
Some College	71	13.42
Associate’s Degree	34	6.43
Bachelor’s Degree	146	27.60
Graduate School	265	50.09
Other/Refused	7	1.33
**Annual Income (USD)**		
0 to 24,999	27	5.10
25,000 to 49,999	53	10.02
50,000 to 74,999	75	14.18
75,000 to 99,999	94	17.77
100,000+	273	51.61
Refused	7	1.32
**Military Branch^a^**		
Army/Army Reserve/Army National Guard	338	63.89
Navy/Navy Reserve	137	25.89
Air Force/Air Force Reserve/Air National Guard	111	20.98
Marine Corps/Marine Corps Reserve	80	15.12
Coast Guard/Coast Guard Reserve	14	2.64
Combat experience, yes	384	72.7
Service-related injury, yes	351	66.6

*^*a*^Participants could select multiple response options for this question.*

Sixty-four percent of veterans had served in the Army, Army National Guard or Army Reserve. Seventy-three percent of veterans in the sample had combat experience, and 66.6% said they had a service-related injury.

### Model Fit Statistics

**FIGURE 1:**
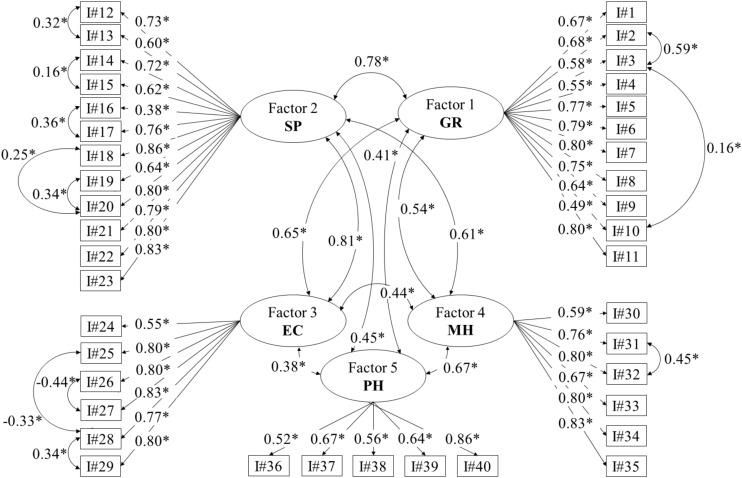
Five-factor model of the Enriched Life Scale with factor loadings and factor covariances. Asterisks indicate statistical significance at *p* < 0.05. SP = sense of purpose, EC = engaged citizenship, PH = physical health, MH = mental health, GR = genuine relationships.

In Table 2, we provide the model-fit statistics for the one-factor, four-factor, and five-factor ELS models. Results showed that the five-factor model was a good fit to the data according to the RMSEA, CFI, TFI, and SRMR statistics, while the one-factor and four-factor models indicated inadequate fit to the data. In addition, since the AIC and BIC values were lower for the five-factor model (AIC = 182,458.85, BIC = 182,890.22) than the one-factor model (AIC = 185,958.80, BIC = 186,300.48) and the four-factor model (AIC = 183,586.04, BIC = 183,953.34), the five-factor ELS model should be preferred. This model is shown in Figure 1. We computed average variance extracted for each latent construct in order to determine the amount of variance explained within each construct by its items and obtained the following results: sense of purpose = 0.55, genuine relationships = 0.46, engaged citizenship = 0.54, mental health = 0.61, physical health = 0.43. Malhotra and Dash (2011) indicated that average variance extracted is “a more conservative measure than CR. On the basis of CR alone, the researcher may conclude that the convergent validity of the construct is adequate, even though more than 50% of the variance is due to error” (p. 702). A full list of the items are described in Angel et al. (2018b) and available from Team Red White Blue (2017).

**TABLE 2:** Model fit statistics for the 40-item Enriched Life Scale (ELS) (*n* = 529).

**Model**	**χ^2^**	***df***	***p*-value**	**RMSEA**	**CFI**	**TLI**	**SRMR**
One-factor model	4,516.42	740	<0.01	0.10	0.64	0.62	0.10
Four-factor model	2,648.67	734	<0.01	0.07	0.82	0.81	0.07
Five-factor model	1,731.51	719	<0.01	0.05	0.90	0.90	0.06

**df*, degree of freedom; RMSEA, root mean square error of approximation; CFI, comparative fit index; TLI, Tucker-Lewis index; SRMR, standardized root mean square residual. The following residual correlations were added to the five-factor ELS model: GR 2 and 3; GR 3 and 10; SP 16 and 17; SP 19 and 20; SP 12 and 13; SP 18 and 20; SP 14 and 15; EC 28 and 29; EC 26 and 27; EC 25 and 28; MH 31 and 32. The five-factor model without residual correlations exhibited the following fit statistics: χ^2^ (730) = 2,317.55, *p* < 0.01, RMSEA = 0.06, CFI = 0.85, TLI = 0.84, SRMR = 0.07, AIC = 183,176.83, BIC = 183,561.22.*

### Internal Consistency Reliability

We assessed internal consistency reliability with Cronbach’s alpha and adopted a criterion of >0.70 to indicate reliability (Nunnaly, 1978). Results showed that the five factors of the ELS exhibited satisfactory internal consistency reliability: genuine relationships, α = 0.90; sense of purpose, α = 0.93; engaged citizenship, α = 0.89; mental health, α = 0.88; and physical health, α = 0.78.

### Convergent Validity and Reliability

Standardized factor loadings for the five-factor ELS model are shown in Figure 1. Results showed that all unconstrained factor loadings within the five factors were statistically significant at an alpha level of 0.05. Furthermore, composite reliability (CR) indices for each factor, which indicated whether items within the same factor measured the same construct (Hair et al., 2008; Thornton et al., 2014), were >0.70: genuine relationships, CR = 0.91; sense of purpose, CR = 0.93; engaged citizenship, CR = 0.89; mental health, CR = 0.88; and physical health, CR = 0.80. Given the criteria outlined in Cole (1987), Hair et al. (2008), and Thornton et al. (2014), results showed that the five factors within the ELS demonstrated convergent validity. We also assessed convergent validity of the five-factor model by calculating Pearson correlation coefficients between scores from the validated five-factor ELS model and factor scores from the M2C-Q, which we hypothesized would be inversely related. Given that all Pearson correlation coefficients were negative and exhibited *p-*values < 0.05, further evidence of convergent validity for the ELS was provided.

### Discriminant Validity

We examined discriminant validity within the five-factor ELS by calculating HTMT among the five factors, using a criterion of <0.85 to indicate discriminant validity (Henseler et al., 2015). Results showed that the HTMT ratios between each of the five factors in the ELS were less than 0.85, providing initial evidence of discriminant validity within the ELS (Table 3). Mean and standard deviations for the final ELS scales ranged from 55.71 (SD = 19.52) for physical health to 75.51 (SD = 16.93) for genuine relationships (Table 3). On a scale of 0 to 100, the mean score on the M2C-Q was 25.43 (SD = 20.51).

**TABLE 3:** Construct validity results for the five-factor ELS model (*n* = 529).

		**Pearson *r***	**Heterotrait-Monotrait Ratios of Correlations (Pearson *r* Correlations)**				
	**M^a^ (SD)**	**M2C-Q^b^**	**GR**	**SP**	**EC**	**MH**	**PH**
GR	75.51 (16.93)	–0.59^∗^	–				
SP	74.92 (17.12)	–0.69^∗^	0.79 (0.72^∗^)	–			
EC	61.52 (21.53)	–0.55^∗^	0.70 (0.62^∗^)	0.80 (0.72^∗^)	–		
MH	70.96 (19.38)	–0.77^∗^	0.51 (0.45^∗^)	0.63 (0.57^∗^)	0.42 (0.38^∗^)	–	
PH	55.71 (19.52)	–0.48^∗^	0.40 (0.33^∗^)	0.47 (0.40^∗^)	0.38 (0.32^∗^)	0.68 (0.57^∗^)	–
ELS Total Score^c^	67.62 (15.17)	–0.78^∗^	–	–	–	–	–

*^∗^Pearson *r* coefficient *p*-value < 0.05. ^*a*^Means and standard deviations for average construct scores.^*b*^M2C-Q Score Mean = 25.43 (SD = 20.51), RMSEA = 0.10, SRMR = 0.07, CFI = 0.82, TFI = 0.80, Chi-Square = 1,746.43, Chi-Square *p*-value < 0.001, α = 0.92. ^*c*^The total Enriched Life Scale (ELS) score is calculated by taking the mean of the five scales (GR, SP, EC, MH, and PH).*

## Discussion

The first aim of this study was to confirm the factor structure of the ELS in veterans not affiliated with Team RWB. Our second goal was to determine if the ELS would have convergent validity with the Military to Civilian Questionnaire, a psychometric measure of reintegration difficulties experienced by veterans.

The results of the CFA indicated that the hypothesized five-factor structure was the most adequate for the ELS, and all items contributed significantly to their corresponding factor: genuine relationships, sense of purpose, engaged citizenship, physical health, and mental health. The model-based reliability for each construct was also excellent. Per the HTMT ratios, the constructs within the ELS were different enough to demonstrate internal discriminant validity.

This finding in a non-Team RWB sample reinforces our initial conceptualization describing veteran transition and as having physical and mental health, people, purpose, and the newly emerged engaged citizenship (continued service) construct as foundational tenets of an enriched life (Angel et al., 2018b). Mean scores and standard deviations were also comparable to those previously reported in the Team RWB sample (Angel et al., 2018b).

The study had several limitations which are noted here, and could be addressed in subsequent research projects. Our sampling approach, which sought to recruit participants via a general request for participation across social media channels and targeted email by partner organizations, may have influenced participation. Based upon the broad solicitation for participation over the course of a year, we cannot tell how many individuals were exposed to a request for participation nor the number of potential respondents the study might have had if all potential respondents had consented to participation. Nevertheless, an achieved sample of over 500 veteran respondents is considered very good for understanding the relationship between latent factors and their constructs, which was the primary goal of the study.

Another potential bias was that we did not assess for the multitude of ways that participants might have been involved in life enriching activities. Based upon the survey recruitment pools, respondents are likely to come from a variety of veteran enriching programs, although we specifically excluded members who self-identified as Team RWB members. Social desirability bias might have influenced the study findings and based upon the recruitment methodology of anonymous participants, we were unable to track them over time. Longitudinal tracking of potential changes in ELS scores is another area of future research. Additionally, our analysis showed that four out of five model fit indices calculated in this study for the M2C-Q indicated poor fit with a one-factor solution. While it was beyond the scope of this paper to investigate an alternative factor structure for the M2C-Q, future studies should consider examining a multi-factor structure for the M2C-Q.

Another limitation of the study is the gender imbalance of participants. While the majority of veteran participants were men, 20% of our study participants were veteran women, twice the number of women veterans comprising the total veteran population in the United States as of 2015 (Office of Data Governance and Analytics, 2017). Understanding the factor structure of ELS for women veterans specifically is an important area for future research. We only tested convergent validity of the ELS with one other measure, which has been done in other studies, such as ones with the PHQ-9 (Cameron et al., 2008) and the SF-6D (Kontodimopoulos et al., 2009). However, future studies should consider testing the convergent validity of the ELS with other multidimensional measures. In addition, as all the scales in the study were self-reported, construct validity of the ELS should be evaluated using different methods in future research, including other types of reporting and additional behavioral measures.

Our results supported our hypothesis that each of the five ELS factors (and ELS total score) were negatively associated with the M2C-Q questionnaire, indicating that veterans who experience greater physical health, mental health, genuine relationships, sense of purpose, and engaged citizenship report fewer reintegration difficulties. This finding has important implications for the implementation of the ELS as a practical assessment tool for veteran health and wellbeing and how it can be integrated into the broader portfolio of clinical assessment tools. The M2C-Q focuses on reintegration challenges. Unlike the battery of other available psychiatric diagnostic and substance misuse instruments administered by the Veterans Health Administration (VHA) and Department of Defense (Patient Health Questionnaire-2, Patient Health Questionnaire-9, Primary Care Post-Traumatic Stress Disorder screen, Alcohol Use Disorders Identification Test-Consumption, Post-Deployment Health Assessment) (Panaite et al., 2018), the M2C-Q is used for screening transition stress related to community integration, personal relationships, self-care, and meaning in life. Consequently, the M2C-Q provides insight into transition-related problems that are neither diagnostic nor reflective of specific mental health related pathology.

While we have very limited visibility of screening instruments implemented in VHA clinical sites and other leading health institutions serving veterans, what we can determine based upon review of publicly available websites via Google search (which may be the only information available to veterans and the layperson community), is that currently veterans seeking information from the VHA website are offered four mental health screening assessments (PTSD screening via the PTSD Check List (PCL); depression screening via the Patient Health Questionnaire-9 (PHQ-9); substance abuse screening via the Alcohol, Smoking and Substance Involvement Screening Test (ASSIST); and alcohol use screening via the Alcohol Use Disorders Identification Test for Consumption (AUDIT-C)^2^. While critical to directing veterans to mental health resources and potentially a starting point for much needed mental health intervention, arguably greater emphasis could potentially be placed on illuminating a broader spectrum of mental health. The ELS’s focus on “what goes right in life,” coupled with the existing strain focused assessments, could help to reframe health assessment aligned to a comprehensive wellness framework, where the underlying message delivered to veteran respondents communicates an expectation of thriving, along with assessment of potential challenges. Doing so potentially helps derail the “victimhood” narrative (Kleykamp and Hipes, 2015), which is perpetuated when health care institutions remain focused on a paradigm of expected brokenness.

By current assessment standards, it is not possible to tell which veterans screen positive for post-traumatic stress, but neverthe-less are still leading a life that they feel is filled with purpose, direction, and shared goals with others. The lived experience of veterans demonstrates that not only can both pathways be possible, but we recommend that communicating both as part of an overall health status is critical, if clinicians are to keep veterans’ holistic health needs at the center of their wellness journeys home. The VHA is leading in so far that they are making strides through the development of their “Whole Health for Life” platform, and the development of their Personal Health Inventory^3^, yet these advances have yet to translate into a publicly available, screening instrument for veterans. The ELS could assist in making that possible.

The ELS has demonstrated tremendous promise for use as a general wellness assessment tool in the civilian community as well. In our preliminary study documenting the ELS’s factor structure, both veteran and civilian versions were nearly identical, with only one item related to sleep falling on the civilian physical health scale and the veteran mental health scale. Our next research steps will be to confirm the factor structure in a sample of civilian community members. We are encouraged about growing evidence that the ELS is a measure of well-being for all people.

## Data Availability Statement

The datasets generated for this study are available on request to the corresponding author.

## Ethics Statement

This study was carried out in accordance with the recommendations of The Institutional Review Board at Syracuse University with electronic informed consent from all subjects. All subjects gave electronic informed consent in accordance with the Declaration of Helsinki. The protocol was approved by the Institutional Review Board at Syracuse University.

## Author Contributions

CA, NA, BY, JP, and MW designed the ELS, conceived the study, and provided conceptual guidance and commentary. CA and RL collected the data. MW and JM analyzed and interpreted the data. CA, MW, RL, and JM contributed to writing the manuscript. All authors reviewed and approved the final version for publication.

## Conflict of Interest

The authors declare that the research was conducted in the absence of any commercial or financial relationships that could be construed as a potential conflict of interest.

## Footnotes

1 https://www.teamrwb.org/reports/annual-report-2018/

2 https://www.myhealth.va.gov/mhv-portal-web/screening-tools

3 https://www.va.gov/PATIENTCENTEREDCARE/resources/personal-health-inventory.asp
